# Seasonal Variations in the Toughness of Leaves: A Case Study Using *Griselinia littoralis*

**DOI:** 10.1093/icb/icae004

**Published:** 2024-03-07

**Authors:** David Taylor

**Affiliations:** Trinity College Dublin, The University of Dublin, Dublin, D02 PN40, Ireland

## Abstract

Potential effects of climate change include greater extremes of temperature and increased severity of storms. Many plants have evolved to resist the challenges of winter (freezing, dehydration, and wind) in a process known as cold hardening. Sensing reducing temperatures, they make structural changes at the cellular level to increase their mechanical resistance and prevent damage. Previous work on this topic, though extensive, has been conducted under laboratory conditions rather than in the field, and while many workers have observed changes to cell wall thickness and composition, which imply increased mechanical strength, few have actually measured strength or any other parameter describing structural integrity. This paper describes experiments on a model system designed to measure the structural integrity of leaf laminae from plants growing naturally in the field over extended periods, allowing seasonal variations to be captured. Standard engineering properties—tensile strength and fracture toughness—were measured for leaves of *Griselinia littoralis* on 19 separate occasions over a 12-month period. Toughness (rather than strength) was found to be the controlling mechanical property. Toughness values were found to change significantly during the year, by more than a factor of 2. Toughness correlated strongly with average daily soil temperature, but with a lag of about 1–2 weeks, suggesting that this is the time needed for structural adjustments to take place. Highest toughness values occurred in winter, confirming cold hardening. Increasing temperature in the spring was associated with decreasing toughness, but in the summer, when highest temperatures occurred, toughness increased again. This apparent “hot hardening” may be a response to dehydration. Results imply that a given leaf is able to both increase and decrease its toughness in response to temperature changes, demonstrating excellent plasticity of response. This case study of a single species establishes a method of reliably measuring changes in a plant’s structural integrity due to cold hardening and other seasonal variations, which may be used to investigate the effects of climate change and other variables.

## Introduction

Cold hardening, also called seasonal acclimation and winter hardening, is the phenomenon by which plants adjust their physical and mechanical properties in order to respond to the potentially damaging effects of low temperature (Smallwood et al. 2002). These effects include dehydration due to reduced fluid transport and damage to cell walls due to freezing of water (Rajashekar and Lafta 1996; Takahashi et al. 2021). In many climates, winter is also a time of increased storm activity, increasing the risk of mechanical damage to leaves, stems, etc. Many plant species have evolved cold hardening, allowing them to survive in areas that frequently experience sub-zero temperatures (Forbes and Watson 1992). This attribute will be a major factor in their ability to survive in the face of climate change, as seasonal temperatures vary from previous norms and storms become more frequent and intense. The phenomenon is also of interest to agriculture, allowing the development of crop species with improved resistance.

### Detection of low temperature

The adaptive mechanisms that underlie cold hardening are still not completely understood, despite considerable research (e.g., Minorsky 1989; Vigh et al. 1993; Örvar et al. 2000; Smallwood et al. 2002; Los et al. 2013). It is suspected that there may be synergisms between those mechanisms that respond to low temperatures and those relating to other stressors such as drought and salinity. An essential first step for the plant is to be able to detect the temperature decrease: One proposed mechanism for this is based on the increase in the viscosity of water in the cell membrane, which has been linked to calcium signaling and the expression of cold hardening genes (Vigh et al. 1993; Los et al. 2013). Cooling causes a rapid transient rise in intracellular calcium (Knight et al. 1996) associated with increased membrane rigidity (Örvar et al. 2000). Several workers have proposed that, in addition to the absolute temperature, the rate of change of temperature is a crucial factor (Minorsky 1989). For example, changes in calcium concentration were found at high cooling rates in the roots of *Arabidopsis*, gradually decreasing to zero at a cooling rate of 10°C/h (Plieth et al. 1999).

### Response to low-temperature damage

Several workers have investigated the damaging changes that occur in the cell membrane as a result of low temperature, and how these are resisted. Though freezing causes water to expand, the cell actually shrinks because water is drawn out. As a result of this dehydration, the membrane becomes folded and may subsequently break (lysis) when the ice thaws and the cell expands again. The degree of damage is often used to characterize resistance to freezing, using an assay that measures leakage of electrolytes (Takahashi et al. 2021). Resistance to lysis has been linked to changes in cell wall thickness and composition (Panter et al. 2020) occurring over periods of several weeks. For example, some workers found changes in cell walls of oilseed rape after 3 weeks at 2°C (Stefanowska et al. 1999). Leaves became thicker during the low-temperature exposure due to cell expansion and cell wall thickening. Cell wall thickening and composition changes, likely to increase stiffness, were observed in winter rye after 7 weeks at low temperature (Griffith and Brown 1982) and also in oilseed rape after 3 weeks of cold exposure.

### Measurement of hardening

These measured changes in cell thickness and composition imply increased mechanical robustness, but this has been measured directly by only a few workers, using various different (and generally non-standard) mechanical property tests. For example, Gomez-Galindo et al. (2004) measured the force needed to slice the tap root in the carrot plant, showing increases over 12 weeks in cold conditions. Rajashekar and Lafta (1996) measured cell wall strength in cells of grape and apple obtained from cell cultures. They recorded the pressure needed to destroy 50% of the cells. After cold hardening for 3–5 weeks at 4°C, this pressure increased by 56% in grape but only 12% in apple (Rajashekar and Lafta 1996). They also stimulated cold hardening in plants of inkberry at 3°C for 6 weeks and showed that the effect could be reversed by “dehardening,” raising the temperature to 20°C for just 1 week. This reversible behavior was also noticed by other workers, who linked it with the formation (and subsequent removal) of extra layers on the cell walls (Steiner et al. 2020).

In summary, previous work has established that temperature changes can be detected very rapidly, within hours or days. Cold conditions cause cell wall damage, which can be mitigated by physical changes that occur over periods of the order of weeks. Only a few workers have conducted experiments to measure changes in mechanical properties, at the cell or organ level, using *ad hoc* experiments for strength measurement rather than the standardized methods used by materials scientists. Almost all work has been conducted on plants of agricultural interest (e.g., fruits and cereals) exposed to specific changes in temperature under laboratory conditions.

### Strength and toughness

Materials scientists and engineers define mechanical parameters that characterize structural integrity, including two properties known as strength and toughness. Strength is defined as the maximum stress (equal to the force normalized by the area over which it acts) that a material can sustain without breaking. However, a brittle material may fail at a stress lower than its strength because it is sensitive to small defects that concentrate stress locally and cause failure by crack propagation. This requires the creation of new surfaces as the crack grows, so, in the broadest sense, toughness can be defined as the difficulty with which surfaces are created.

**Fig. 1:**
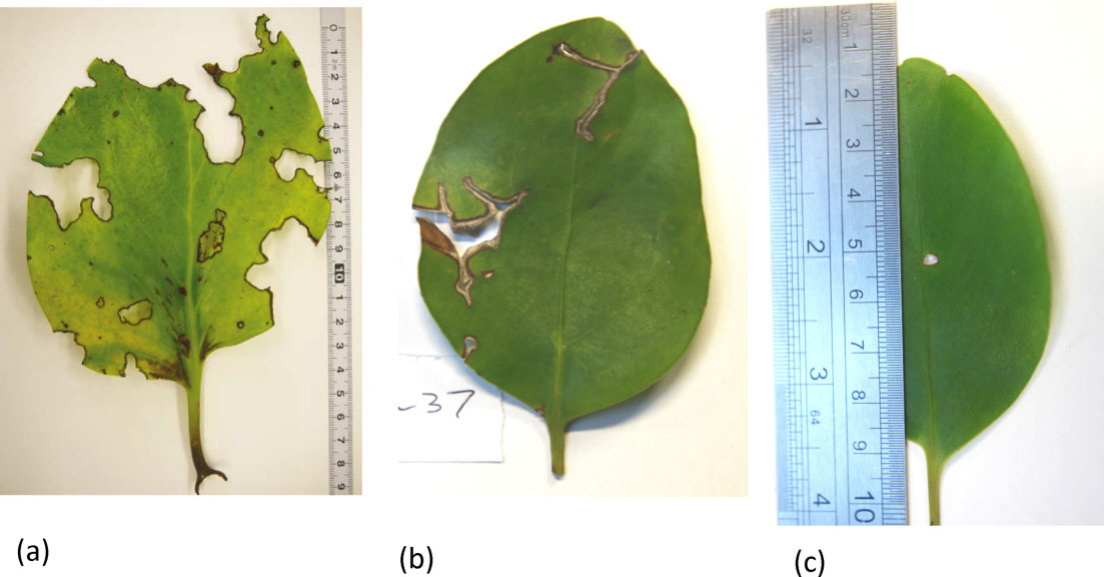
Examples of naturally occurring defects in leaves: (a) *Griselinia lucida* damaged by insect herbivory (Bryan et al. 2011), reproduced with permission; (b, c) *G. littoralis* (present work, causes unknown).

Strength and toughness are important properties for leaves, for two reasons: first, in relation to herbivory, which involves the fracture of the leaf into pieces small enough for consumption, whether by large mammals or by insects (Vincent 1990); and second, leaves may break as a result of mechanical forces, including wind, precipitation, and impacts from animals. If toughness is low, then the force needed to break the leaf will decrease when defects are present, such as holes or notches caused by herbivory (Bryan et al. 2011) or by diseases. Figure 1 shows some examples of such defects.

### Measurement of leaf toughness

In their review Read and Stokes noted that, given the wide variety of mechanical tests applied to leaves by different workers, it was difficult to distinguish between measurements of toughness and measurements of strength (Read and Stokes 2006). Toughness tests previously performed include penetration with a blunt or sharp object, cutting with a scissors or blade and the notch tensile test (Lucas 1991; Rowe II and Potter 1996; Sanson 2006; Volaire et al. 2018).

The notch tensile test is the preferred method for toughness measurement for engineering materials, described in several internationally recognized standards (e.g., ASTM E1820, The American Society for Testing and Materials). The test involves applying a gradually increasing force to a sample of the material which already contains a crack. The stress needed to cause the crack to propagate is used to calculate a property known as the fracture toughness, *K*_c_, given by following equation:


(1) \begin{eqnarray*}
{{K}_\mathrm{ c}} = Q{{{\mathrm{\sigma }}}_{\mathrm{f}}}\surd \left( {{\mathrm{\pi }}a} \right).
\end{eqnarray*}

Here, σ_f_ is the stress at which the specimen fractures, conventionally calculated as the “nominal stress,” i.e., the stress that would exist if the crack were not present; *a* is the length of the crack; and *Q* takes a value that depends on various factors such as the size and shape of the specimen and the type of applied force. Theoretically, a perfectly sharp crack (having zero root radius) is required, but in practice, all real cracks have finite radii, and it is often possible to use a cut or machined notch instead of a crack, provided certain criteria are fulfilled.

**Fig. 2:**
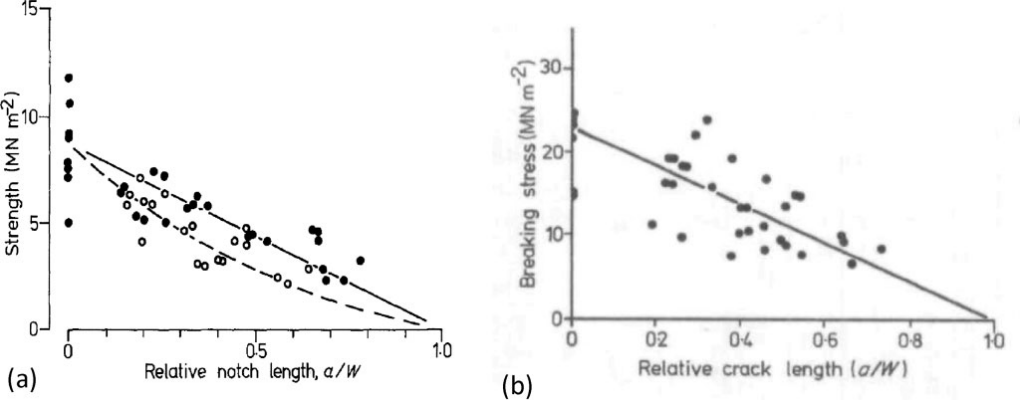
Examples of test data from the literature (fracture stress versus normalized notch length) for leaves of rye-grass *Lolium perenne*: (a) (Vincent 1982), the different symbols indicate different specimen types; and (b) (Vincent 1983), reproduced with permission.

The great majority of work on leaf toughness has used the scissors, blade, or penetration methods but a small number of papers have reported *K*_c_ values obtained from notch tensile tests (Vincent 1982, 1983, 1991; Vincent and Gravell 1986; Lucas 1991; Toole et al. 2000). Figure 2 shows an example of previous results, plotting the stress to cause failure as a function of the ratio between the crack length and specimen width *a*/*W*. When *a*/*W* is zero, there is no crack, and so the failure stress is equal to the ultimate tensile strength of the material, σ_u_. For any finite crack length, the stress is σ_f_, from which the toughness can be calculated (Equation 1). A line is drawn on this plot from (0,σ_u_) to (1,0). If the points lie on this line, then the notch has had no effect on the strength except in so far as it has removed some material and therefore reduced the cross section in that region. In this case, the integrity of the leaf is determined by its strength, not by its toughness. If the data points fall below the line, then the specimen displays notch sensitivity, and the toughness can be calculated. In Fig. 2a, the two different symbols here indicate two different datasets. For one of these, and also for the results in Fig. 2b, the points are scattered equally above and below the line, indicating that strength is the important parameter. The other dataset in Fig. 2a lies below the line, implying that toughness is important here.

The results in Fig. 2 show quite a lot of scatter. For example, the analysis of the results in Fig. 2a shows a COV (coefficient of variance, equal to the standard deviation divided by the mean) of 0.25. Data from other workers also showed considerable scatter (Toole et al. 2000). The most consistent data available are that from Lucas et al. (1991) who tested leaves from *Calophyllum inophyllum*. From these publications, we can conclude that leaves from different species vary as to whether toughness or strength is relevant to their integrity and that scatter is considerable in some studies, whether due to natural variations in the specimens or to experimental inaccuracies. Lucas et al. also compared the notched tensile test with other ways to measure toughness (scissors and penetrometer) finding significant differences in results. These other tests measure the energy required to create a certain amount of new surface, expressing the toughness as a parameter *G*_c_ with units of Joules per square meter. *G*_c_ and *K*_c_ are simply related to each other through the material’s elastic properties, so one parameter can be calculated from the other. While all toughness tests create new surfaces, the blade and penetrometer tests impose a certain surface by cutting, while the notch test allows the crack to propagate freely, choosing (to some extent) the path of least resistance, so some differences in the results should be expected.

In summary, though standard methods exist for the measurement of toughness and strength, and though these have been applied to plants previously, no such measurements have been used to characterize cold hardening. Most cold hardening experiments have been conducted under laboratory conditions rather than in-field, using non-standard methods to determine (or infer) mechanical fitness, and the focus has been very much on the changes induced by temperature reduction with relatively little attention paid to the subsequent restoration of properties when temperatures rise again. Therefore, the objectives of the present work were as follows:

- To measure strength and toughness in leaves, commenting on the accuracy and suitability of these measurements to characterize the leaf’s mechanical properties.
- To conduct measurements throughout a 12 month period to attempt to detect cold hardening and other seasonal changes.
- To investigate correlations between toughness and ambient temperature.

## Methods and materials

The species chosen for testing was *Griselinia littoralis*, a dicotyledon, evergreen shrub found very extensively in temperate climates, commonly known as *Griselinia*. Cold hardening has not previously been investigated in this species. It was chosen as a model, being readily available and having leaves with a homogeneous structure and a size suitable for making specimens for mechanical testing.

**Fig. 3:**
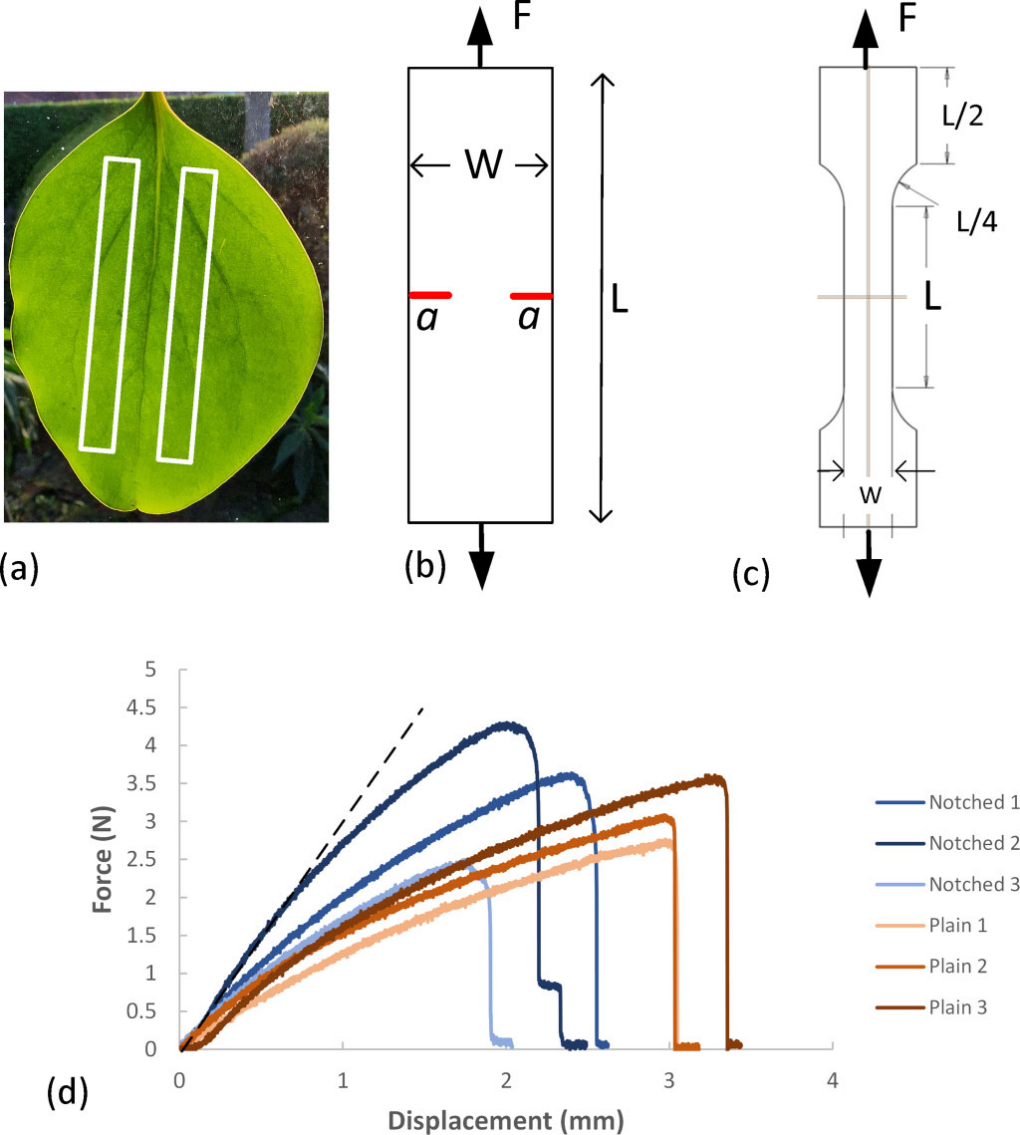
(a) A leaf showing the pattern of veins and the location of two rectangular strips from which specimens were made. (b) Notched specimen geometry for the toughness tests. (c) Plain (dogbone) specimen geometry for the strength tests. (d) Typical force/displacement results for notched and plain (dogbone) specimens. A straight line has been fitted to one of the curves to demonstrate their downward curvature.

Leaf samples were taken from the same group of plants on 19 dates, from March 14, 2022 to March 10, 2023. Leaves were removed from the plant around 8 am each day, stored in a sealed container to prevent water loss, and tested within 3 h. Relatively large leaves (typically 100 mm long and 75 mm wide) were chosen for consistency and to facilitate the cutting out of specimens. For the determination of *K*_c_, tests were carried out using double-edge notched specimens (see Fig. 3). Leaves of this species have a well-defined primary vein running along the central axis and some prominent secondary veins near the base (see Fig. 3a). Preliminary tests showed that these veins could affect the results, preventing or hindering crack propagation. So specimens were cut with their long axis parallel to the primary vein, lying close to but not including the vein (Fig. 3a), and notches were placed with their tips remote from secondary veins. Specimen width *W* and thickness *t* were measured using digital calipers. Sharp notches were cut with a scalpel and their lengths were measured using a microscope with a graduated eyepiece. The toughness of some leaves is known to be dependent on sample orientation (Lucas 1991); some preliminary testing (not reported here) showed that in this species the toughness is not orientation dependent, but nevertheless in this work the same orientation was used throughout. Some specimens were cut with no notch and with a waisted central section and wider ends (known as a dogbone shape [Fig. 3c]) for testing to find the material’s tensile strength. A minimum of six leaves were tested on each date, providing 12 samples of which, normally, six were used for tensile testing and six for strength testing. On four occasions, the strength testing was omitted in order to carry out other investigations (not reported here).

Tests were carried out on an Instron 3366 mechanical testing machine (Instron, High Wycombe, UK). Samples were attached to metal clamps and pulled at a rate of 1 mm/min while monitoring force continuously until failure. Figure 3d shows typical force/displacement traces for both plain (i.e., dogbone) and notched specimens. The lines are relatively straight until close to failure, indicating largely elastic behavior, which is a requirement for the validity of this test. There is some downward curvature indicating viscoelasticity, which does affect the resulting toughness value (by consuming energy), but this has been considered in previous work and estimated to create only a small error, around 13% (Vincent 1990).

All failures occurred from the notches or within the gauge length for the unnotched specimens. Cracks propagated straight across the specimen width. Cracks were seen to begin propagating at or just before the peak force, so this was defined as the failure force *F*_f_. The failure stress σ_f_ for use in Equation (1) was calculated as


(2) \begin{eqnarray*}
{{{\mathrm{\sigma }}}_{\mathrm{f}}}{\mathrm{ = }}{{F}_{\mathrm{f}}}{\mathrm{/}}Wt.
\end{eqnarray*}

The value of *Q* in Equation (1) was obtained from the literature (Murakami 1987):


(3) \begin{eqnarray*}
Q &=& 1.122 - 0.154\left( {a/W} \right)\ + \ 0.807{{\left( {a/W} \right)}^2}\\
&&\quad - 1.894{{\left( {a/W} \right)}^3} + \ 2.494{{\left( {a/W} \right)}^4}.
\end{eqnarray*}

Most tests were conducted with specimen width *W* = 11 mm and crack length *a* = 3 mm, but on some occasions tests were also conducted with other widths (ranging from 5 to 20 mm) and other crack lengths (1.0–7.5 mm): results showed no significant effect of these variables.

Ambient temperature values were obtained from a database compiled by Met Eireann, the Irish meteorological service, available online at met.ie. Measurements taken at Dublin Airport (a distance of 19 km from the sampling site) were used. The available daily temperature values were maximum air temperature, minimum air temperature, and average soil temperature. The temperature value chosen for analysis was average daily soil temperature (measured at a soil depth of 10 cm). However, the soil temperature correlated quite closely to the average of the two air temperatures, with an *R*^2^-value of 0.81, suggesting that average daily air temperature could equally well have been used if it had been available.

Statistical analysis was conducted to compare results from experimental groups and to validate theoretical models. Non-parametric tests (Kruskall–Wallis and Mann–Whitney) were used for comparing test groups (i.e., results from individual dates) because the group sizes were small and not always normally distributed. ANOVA and *t*-test were used to assess the accuracy of the regression modeling applied to the entire dataset of 123 results. The chosen a significance level was *P* = 0.05.

## Results

**Fig. 4:**
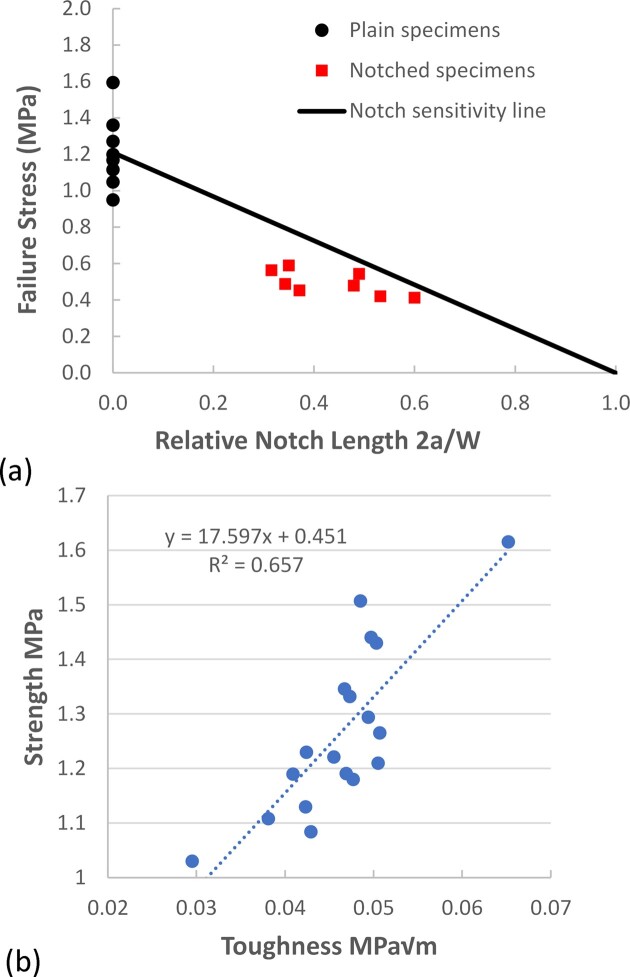
(a) Failure stress as a function of normalized crack length 2*a*/*W* for eight tests conducted on the same day. (b) Correlation between mean strength and mean toughness (paired samples from the same leaf) for all dates on which both properties were measured.

**Fig. 5:**
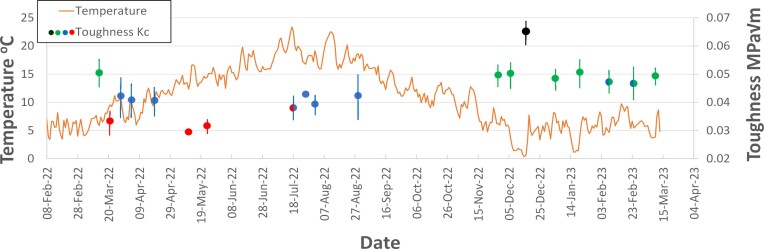
Toughness and temperature as a function of the date of the measurement. Bars on the toughness results show standard deviation. Different colors indicate statistical significance in the toughness results (*P* < 0.05), corresponding to groups listed in Table 1, with red = A, blue = B, green = C, and black = D. Three points fall into two groups (red/blue = A/B and blue/green = C/D).

Numerical results are summarized in Table 1 and presented in Figs 4 and 5. Measurements of toughness *K*_c_ obtained from sets of specimens on the same day showed remarkably little scatter, with COV values from 0.044 to 0.163, averaging 0.10. Measurements of strength σ_u _showed similar accuracy with the same average COV of 0.10. The results show less variability than most of the previously published data on leaves.

**Table 1:** Results showing toughness (*K*_c_, in MPa m^1/2^) and strength (in MPa), with standard deviations.

Date	*T*_9*−*5_	Group	*K*_c_	SD of *K*_c_	σ_υ_	SD of σ_υ_
14/03/2022	4.80	C	0.0505	0.0048	1.21	0.2
21/03/2022	6.91	A	0.0334	0.0045		
28/03/2022	8.80	B	0.0423	0.0069	1.13	0.13
04/04/2022	10.09	B	0.0409	0.0059	1.19	0.16
19/04/2022	8.79	B	0.0407	0.0048		
11/05/2022	13.23	A	0.0295	0.0013	1.03	0.08
23/05/2022	15.28	A	0.0317	0.0024		
18/07/2022	20.20	A/B	0.0381	0.0046	1.108	*0.043*
26/07/2022	21.48	B	0.0429	0.0013	1.084	0.079
01/08/2022	18.00	B	0.0394	0.0033		
29/08/2022	18.46	B	0.0424	0.0089	1.23	0.122
28/11/2022	5.66	C	0.0497	0.0037	1.44	0.148
06/12/2022	6.75	C	0.0503	0.0048	1.43	0.14
16/12/2022	2.08	D	0.0652	0.0041	1.615	0.22
04/01/2023	5.01	C	0.0485	0.0037	1.507	0.048
20/01/2023	5.54	C	0.0507	0.0051	1.265	0.138
08/02/2023	6.50	B/C	0.0473	0.0040	1.332	0.152
24/02/2023	8.50	B/C	0.0467	0.0059	1.346	0.169
10/03/2023	6.25	C	0.0494	0.0030	1.294	0.167

Results in the same group (A, B, C, or D) have *K*_c_ values significantly different from those in all other groups (*P* < 0.05), except for three results (designated A/B or B/C), which fall within two groups. Also shown is the temperature function, which best correlates with the toughness data: *T*_9*−*5_, in °C.

Figure 4a shows an example of test results in the form used by previous workers (Fig. 2), in which the failure stress is plotted as a function of the ratio between total crack length and specimen width, 2*a*/*W*. All of the data points fall below the line, indicating notch sensitivity and allowing toughness to be calculated. Values of toughness and strength showed quite good correlation (Fig. 4b) with a linear relationship and an *R*^2^-value of 0.66 (statistically significant at *P* < 0.05).

Significant changes were found for measurements made on different days over the 12 month period. Figure 5 shows toughness and temperature as a function of the testing date. Toughness values are indicated using data points of four different colors, representing increasing toughness in the order: red, blue, green, and black. Points having a given color are significantly different from points of all other colors (*P* < 0.05). The only exceptions are three points that fall within two groups, so these are shown with two colors, red/blue or blue/green. This information is also given in Table 1, where the groups are assigned a letter A, B, C, or D. Toughness varied during the year by more than a factor of two. Lowest toughness values, around 0.03 MPa m^1/2^ occurred during the springtime (Fig. 5b). During the winter months, toughness was high, typically around 0.05 MPa m^1/2^ but rising as high as 0.65 MPa m^1/2^ on one occasion.

### Analysis: correlation between toughness and temperature

Some explanations for this toughness variation can be ruled out. The procedure for harvesting and testing samples was consistent and designed to avoid any effects of sampling time and dehydration after sampling. The environmental conditions in the laboratory, temperature, and humidity, were not monitored during this work but have been recorded during other projects and found to be quite constant at around 19 ± 2°C and 35 ± 5% relative humidity, so it is unlikely that toughness would be affected by testing conditions. Specimen dimensions (*W, t*, and *a*) were found to have no effect on measured toughness.

The data were interrogated with the aim of finding correlations between toughness *K*_c_ and temperature. Initial examination of the data gave hints as to possible correlations. In particular, examination of the behavior in the March/April 2022 and in December 2023 suggested that abrupt changes in toughness were associated with obvious changes in temperature in the days immediately prior to the test date. Various functions of temperature were considered. A general *n*th order polynomial was considered, of the form:


(4) \begin{eqnarray*}
{{K}_\mathrm{ c}} = \ {{a}_0} + \ {{a}_1}f\left( T \right)\ + \ {{a}_2}f{{\left( T \right)}^2} + \ldots .{{a}_n}f{{\left( T \right)}^n}.
\end{eqnarray*}

Here, *f(T)* is some function of temperature and *a*_0_, *a*_1_. . .*a*_n_ are constants. It was hypothesized that toughness was determined by the temperature over some period of time preceding the day in question. To investigate this hypothesis, various functions *f(T)* were considered, of the form:


(5) \begin{eqnarray*}
F\left( T \right)\ = \ T_{i{-}j}.
\end{eqnarray*}

Here, *T_i−j_* is the average temperature over the period from *i* days to *j* days before the day in question. So, for example, *T*_5*−*3_ represents the period between 5 days and 3 days prior to sampling, while *T*_1*−*1_ represents the temperature on the day immediately prior to sampling.

A range of values of *i* and *j* were considered, covering a period of 3 weeks. An equation of the form of Equation (4) (with a chosen value of *n*) was fitted to all the data points by minimizing the squares of the residuals, the goodness of fit being expressed by the *R*^2^ parameter. It was found that the optimum function was *T*_9*−*5_: the average temperature in the period between 9 and 5 days before the day in question.

Different orders of polynomial, i.e., different values of *n* in Equation (4), were considered. A value of *n* = 0 amounts to assuming that *K*_c_ is constant, equal to its average value from all tests, which was 0.0435 MPa m^1/2^. This is the null hypothesis—that toughness is not affected by temperature—against which other predictions can be compared. Table 2 presents results for *T*_9*−*5_, showing best-fit values of the constants *a_i_* values of *R*^2^ and also values of *P* obtained by comparing residuals with those for the null hypothesis, for *n* values in the range 1–3. The *P*-values demonstrate that all predictions are better than the null hypothesis, confirming that toughness does change with temperature. Both *R*^2^ and *P* greatly improve on going from *n* = 1 to *n* = 2 but do not change on increasing to *n* = 3. Thus, the second-order polynomial was found to be optimal, giving the same predictive accuracy as higher orders but using fewer parameters, giving the following relationship:


(6) \begin{eqnarray*}
{{K}_\mathrm{ c}} &=& 0.074215 - 0.005494\left( {T_{9{-}5}} \right)\\
&&\quad + 0.000190{{\left( {T_{9{-}5}} \right)}^2}.
\end{eqnarray*}

**Table 2:** Results from the model analysis for polynomial models of different order *n*, showing model constants *a _i_ , c*orrelation coefficients *R*^2^, and statistical significance (*P-*values compared to the null hypothesis: *n* = 0).

*n*	0	1	2	3
*a*_o_	0.0435	0.052954	0.074215	0.075477
*a*_1_	-	−0.000866	−0.005494	−0.005954
*a*_2_	-	-	0.000190	0.000273
*a*_3_	-	-	-	−1.3 × 10^−6^
*R* ^2^	-	0.374	0.7582	0.7588
*P*	-	0.026	6.2 × 10^−6^	5.7 × 10^−6^

**Fig. 6:**
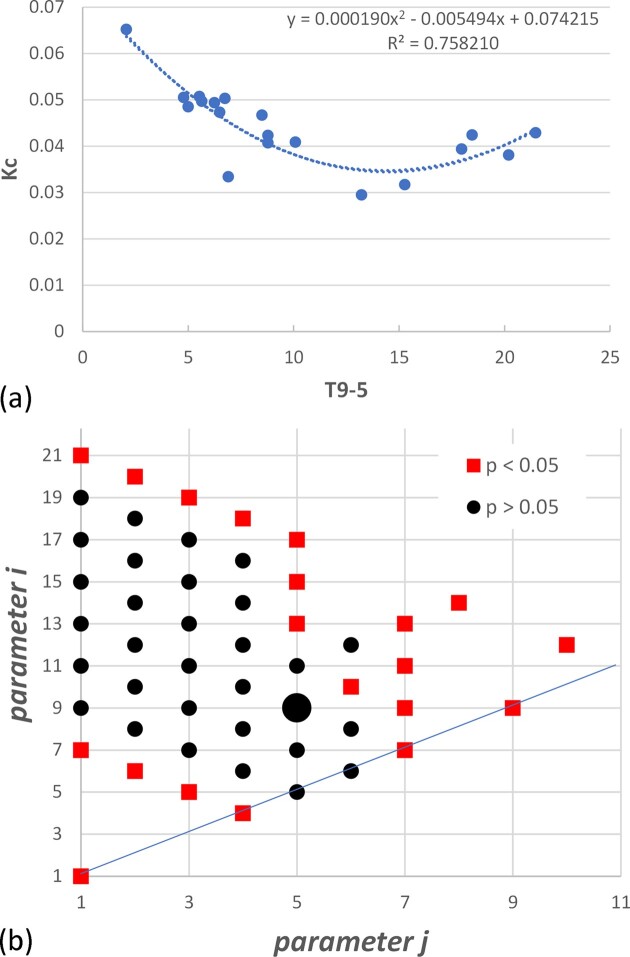
(a) Toughness plotted against *T*_9*−*5_: the average temperature over the period 9–5 days before sampling. This temperature function gave the best correlation. The line shows a quadratic fit to the data. (b) Results from a statistical analysis of regression models: the parameters *i* and *j* in the function *T_i−j_* are plotted. The large black dot indicates *T*_9*−*5_: other black dots indicate functions that are not statistically different from *T*_9*−*5_ (*P* > 0.05): red squares indicate functions that give significantly worse correlations (*P* < 0.05). Since *i ≥ j*, values below the blue line are not possible.

This function is shown in Fig. 6a. It is notable that it is not a monotonic decrease of *K*_c_ with increasing temperature as might have been expected, but instead curves up at the highest temperatures due to the negative value of the squared term. This will be discussed further below.

Different functions with different *i* and *j* values were evaluated over a wide range. Though *T*_9*−*5_ gave the best correlation, some other functions gave correlations almost as good. The results are shown in Fig. 6b, which is a plot of *i* against *j* showing which functions gave fits that were statistically different from that obtained for *T*_9*−*5_.

## Discussion

This work has shown that tensile strength and fracture toughness can be measured repeatably and precisely for leaves, at least from this particular species, using standard materials science methods. Brittle materials tend to display a lot of variability in strength because they are sensitive to small inherent defects, but this was not the case here: The COV values for strength were the same as those for toughness. Some scatter inevitably arises from experimental procedures; in this case, the greatest error likely arises during measurement of thickness due to local variations and because the calipers tend to compress the soft material. It is estimated that errors of the order of 0.02 mm arose when measuring *t*, which had an average value of 0.45 mm, so this implies a 4% error from this measurement alone.

It was also shown that strength and toughness are strongly correlated (Fig. 4b). This suggests that the material belongs to a class known as “quasi-brittle,” which also includes fiber composite materials such as carbon fiber reinforced polymers, which display similar characteristics. This finding is very useful as it justifies the decision of Read and Stokes (2006) mentioned above to consider strength and toughness as equivalent parameters and implies that a variety of simple tests (such as penetrometers, which are easy to use in the field) can provide useful information, at least of a comparative nature.

Strength and toughness can be combined to define a parameter *L*, as follows:


(7) \begin{eqnarray*}
L = \ \left( {1/{\mathrm{\pi }}} \right).{{\left( {{{K}_{{\mathrm{ c}}}}/{{\sigma }_{\mathrm{u}}}} \right)}^2}.
\end{eqnarray*}

*L* is known as the critical crack length; if a crack greater than this length is present, then the fracture stress will be reduced below the material’s tensile strength. In the present case, its average value is 0.42 mm. Cracks as small as this (and other defects such as those shown in Fig. 1) will almost always be present on a leaf, so in practice, the stress to failure will be limited by the material’s toughness and by the size of the largest defect present. Thus, while toughness and strength are correlated (Fig. 4b) making them, in a sense, equivalent parameters, knowledge of the toughness value is important because it alone determines whether or not the leaf will fracture.

Several interesting correlations were found between toughness and temperature. The most obvious of these is that toughness increased during the winter months, demonstrating that cold hardening occurs in this species. There is a corresponding reduction in toughness during the rest of the year and a strong correlation to temperature with the best fit occurring with *T*_9*−*5_. Some other functions, *T_*i − j*_* also gave good fits that were not statistically different (see Fig. 6b). To obtain a good correlation, some data from times greater than 4 days and less than 6 days had to be included; times up to 19 days could be included in the average provided earlier days were also included. So, while there is some uncertainty about the time required, the results suggest that it takes the plant about 1–2 weeks to alter its toughness in response to changes in temperature. This time period is in broad agreement with the laboratory-based studies described above, in which significant changes were observed after cold exposures of 1–7 weeks (Griffith and Brown 1982; Rajashekar and Lafta 1996; Stefanowska et al. 1999; Panter et al. 2020), despite the fact that some measurable quantities (calcium concentration, gene expression) occur much more quickly (Smallwood et al. 2002).

The leaves used in the present work were relatively large, among the largest that the plant produces. Such leaves remain on the plant for several months at any time of the year. So, the low toughness values during the spring (March–May) would have been measured on leaves that had been present throughout the winter, when they would have had higher toughness, and the very high toughness value measured on one date in December was immediately followed by several lower values. This would seem to suggest that not only can leaves increase their toughness in a classic cold hardening process, they can also decrease toughness when spring comes, showing the same ability to “deharden” as demonstrated by other workers (Rajashekar and Lafta 1996). Presumably, there is some advantage to being able to do this, rather than continuing with a high toughness in these old leaves.

There was also an increase in toughness recorded at the very highest temperatures, during the summer months, when *K*_c_ values rose by about 33% above their springtime low values. One possible explanation for this is that the plant was protecting itself from dehydration, a significant stressor during hot weather. This effect could be called “hot hardening.” I did not find any previous work specifically referring to it, which suggests that it merits further study. Dehydration affects the mechanical properties of all biological materials, tending to reduced their stiffness, strength, and toughness. Additionally, in plants, structural stiffness will be reduced due to loss of turgor pressure. This will affect the plant’s response to storms in complex ways, which merits further study.

When analyzing any dataset it is interesting to look at outliers, and these results showed one in particular, which can be seen in Fig. 5b, occurring on the second measurement date. After a high initial toughness of 0.051 MPa m^1/2^ on March 14th, toughness fell rapidly to 0.033 MPa m^1/2^ 1 week later. In all the attempted correlations, this point invariably lies below the prediction line by a considerable margin (see Fig. 6a); i.e., the toughness on that day was less than expected based on the temperature. Examining the temperature record in Fig. 5, one can see that before March 14th the temperature was low and fairly constant, representing typical winter temperatures at this location. From then until March 24th, the temperature rose quickly, increasing by 5°C in 10 days. Previous work (discussed earlier) has often emphasized the importance of the rate of change of temperature, which several workers have concluded is even more important than the absolute temperature. One can speculate that this exceptional drop in toughness was triggered by the plant’s response to a rapid temperature rise following the constant low winter temperatures. However, there were other periods during the year when temperatures rose rapidly and these did not lead to similar drops in toughness. An attempt was made to include temperature gradient d*T*/d*t* into the fitting procedure, using functions of the form


(8) \begin{eqnarray*}
T_{i{-}j}\ + g{\rm d}T/{\rm d}t_{i{-}j},
\end{eqnarray*}

where *T*_*i − j*_is the average temperature during days *i* to *j* and d*T/*d*_i − j_* is the average temperature gradient during the same period, *g* being a constant that allows for different weightings of the two factors. The full dataset was interrogated, but no correlations could be found that were better than the ones obtained using *T_*i − j*_* only. The fit to this particular data point could only be improved at the expense of other data points for which high gradients occurred. So, this outlier remains interesting and worthy of further study: it may perhaps be an effect that only occurs at this particular time of the year, at the onset of spring.

The physical mechanisms controlling toughness were not investigated in this study. Future work could involve detailed anatomical studies at different size scales to detect changes in structure and composition, as has been usefully carried out to investigate other plants’ responses to changing mechanical conditions (e.g., Paul-Victor and Rowe 2011). Some previous work noted changes in the thickness of the whole leaf lamina (Panter et al. 2020), but this did not occur here: measured thickness did not vary significantly with *K*_c_ (*P* = 0.11 compared to the null hypothesis), so it is likely that all significant changes occurred at the cell wall level.

Climate change effects—higher or lower seasonal temperatures and stronger storms—were not specifically considered in this work. However, the methodology developed here forms the basis for future studies addressing these issues. Computer modeling and wind tunnel experiments could be used to investigate the effect of wind speed on leaf stress. The results could be fed into fracture mechanics models to investigate crack propagation in both intact and damaged leaves, leading to predictions of the risk of fracture and leaf loss. Plants could be grown in controlled environments to investigate toughness variations under conditions of controlled temperature change, including the simulation of more extreme climatic conditions.

This work considered only one species, being conceived as a case study to develop the methods for testing and analysis. Leaf laminae in this species are relatively easy to study because they show no anisotropy and contain relatively few prominent veins, factors that certainly will affect toughness measurement in other species. Even here, though, veins can play a role in hindering crack propagation, an effect that merits further study. The work was confined to leaves of relatively large size (about 100 mm), the oldest that were present. However, a few tests were carried out on smaller, younger leaves (65 mm), which had the same toughness, suggesting that size/age is not a major controlling factor. It would be interesting to further investigate the relationships between age, size, and toughness.

## Conclusions

- Strength and toughness can be measured precisely and repeatably, at least for this particular species of leaf.
- For *G. littoralis*, toughness is sufficiently low to be the determining factor controlling leaf fracture, so its accurate measurement is important.
- Cold hardening occurs in this species: toughness varied by more than a factor of 2 between winter and spring. Some evidence suggests that individual leaves may be able to decrease, as well as increase, their toughness.
- The nonlinear correlation between toughness and temperature suggests a second toughening process, in response to high summer temperatures, possibly to combat dehydration.
- Correlations with ambient temperature suggest that it takes about 1–2 weeks for the plant to adjust its toughness. Some anomalous results suggest a possible role for temperature gradient.

## Supplementary Material

icae004_Supplemental_File
